# Hsa-miR-494-3p attenuates gene HtrA3 transcription to increase inflammatory response in hypoxia/reoxygenation HK2 Cells

**DOI:** 10.1038/s41598-021-81113-x

**Published:** 2021-01-18

**Authors:** Qian Gong, Zhi-ming Shen, Zhe Sheng, Shi Jiang, Sheng-lin Ge

**Affiliations:** Department of Cardiovascular Surgery, The First Affiliated Hospital of Anhui Medical University, Hefei, 230022 Anhui China

**Keywords:** Cardiology, Diseases, Nephrology, Pathogenesis

## Abstract

The occurrence of cardiac surgery-associated acute kidney injury (CSA-AKI) increases hospital stay and mortality. MicroRNAs has a crucial role in AKI. This objective of the current study is to explore the function of hsa-miR-494-3p in inflammatory response in human kidney tubular epithelial (HK2) cells with hypoxia/reoxygenation. According to KDIGO standard, patients after cardiac surgery with cardiopulmonary bypass were divided into two groups: AKI (n = 10) and non-AKI patients (n = 8). HK2 were raised in the normal and hypoxia/reoxygenation circumstances and mainly treated by overexpression ofmiR-494-3p and HtrA3. The relationship between miR-494-3p and HtrA3 was determined by dual-luciferase reporter assay. Our result showed that Hsa-miR-494-3p was elevated in the serum of patients with CSA-AKI, and also induced in hypoxic reoxygenated HK2 cells. Hsa-miR-494-3p also increased a hypoxia-reoxygenation induced inflammatory response in HK2 cells. Moreover, as a target gene of miR-494-3p, overexpression of HtrA3 downregulated the hypoxia-reoxygenation induced inflammatory response in HK2 cells. Overexpression of hsa-miR-494-3p-induced inflammatory response was inhibited by overexpression of HtrA3. Collectively, we identified that hsa-miR-494-3p, a miRNA induced in both circulation of AKI patients and hypoxia-reoxygenation-treated HK2 cells, enhanced renal inflammation by targeting HtrA3, which may suggest a possible role as a new therapeutic target for CSA-AKI.

## Introduction

Acute kidney injury (AKI) is a common and serious complication of hospitalized patients, which results in oliguria, anuria and elevated creatinine, and apparently increases the mortality of hospitalized patients^1–3^. Cardiac surgery with cardiopulmonary bypass (CPB) is the second most common cause of AKI after sepsis^4^. At present, CPB is considered to be the main cause of AKI after cardiac surgery, and ischemia–reperfusion injury is considered the main pathway of CPB associated-kidney injury^5–8^. Cardiac surgery-associated acute kidney injury (CSA-AKI) patients are the ideal AKI study population because the time of damage is known, and CPB provides a non-physiological condition of ischemia and hypothermia, it is a high risk factor for kidney damage.

MicroRNAs (miRNAs) are small non-coding RNAs with a length of about 20–24 nucleotides. By binding to the 3′-non-coding region of targeted mRNA^9^, miRNAs inhibit the translation of targeted proteins and achieve negative regulation of gene expression at the post-transcriptional level^10^. MiRNAs is the most frequently studied non-coding RNA for AKI^11^. Current studies on animal models and some types of AKI, such as AKI after ischemia–reperfusion injury^12^, kidney transplantation^13^ and application of nephrotoxic drugs^14^, have accumulated evidence that a variety of miRNAs may be involved in the pathogenesis of AK^15–17^, showing its potential clinical value, providing a new way to study the molecular mechanism of CSA-AKI. MicroRNAs of CSA-AKI is secreted into circulation or urine, so can be used as highly sensitive and specific biomarkers to study the occurrence, development and prognosis of CSA-AKI^18^. The study on upregulation of miR-494 has been identified as an oncogenic miRNA and is associated with poor prognosis in several types of human cancer^19–21^. However, miR-494 is found downregulated and suppressed cell proliferation in some cancer cells^22–24^. At present, upregulation of hsa-miR-494-3p promotes acute kidney injury by negatively regulating ATF3 protein synthesis, because ATF3 plays a protective role in renal ischemia–reperfusion injury^25^. However, the network regulation of hsa-miR-494-3p on renal injury is the same complex as on cancer. It is unclear whether hsa-miR-494-3p affects the severity of kidney injury by regulating other proteins, so we try to further study hsa-miR-494-3p on this point.

## Results

### Hsa-miR-494-3p is elevated in the serum of patients with CSA-AKI

**Figure 1:**
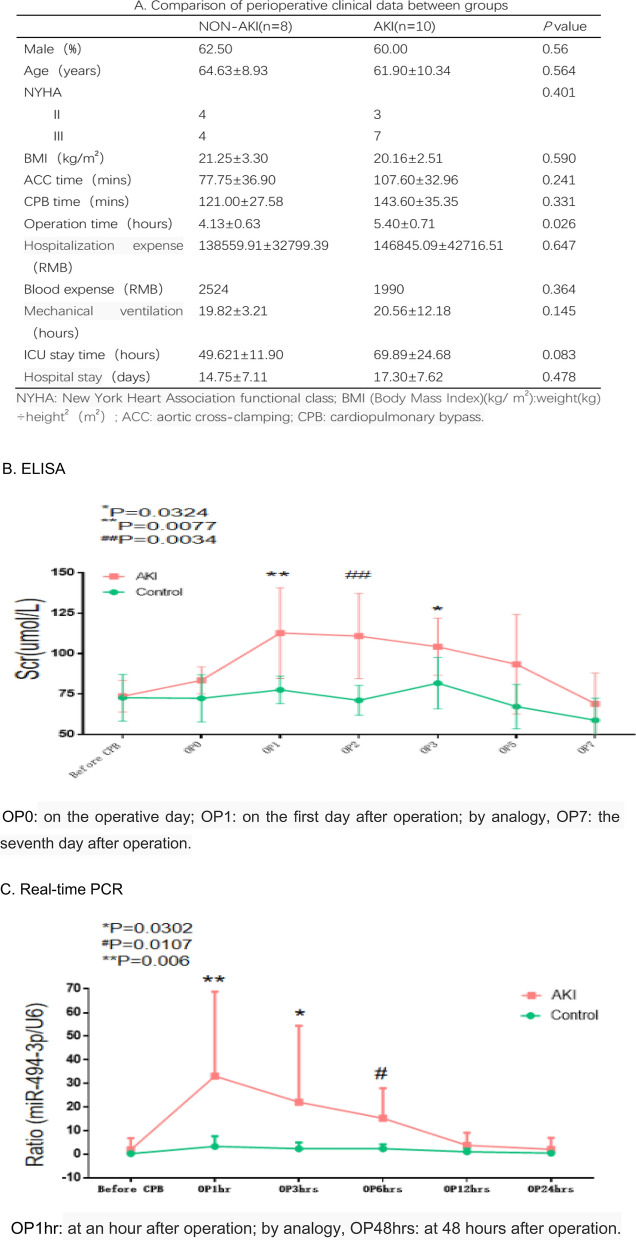
Comparison of perioperative clinical data, serum creatinine and hsa-miR-494-3p between two groups with cardiopulmonary bypass. (**A**) Comparison of perioperative clinical data between groups; (**B**) According to KDIGO standard, Elisa data show the difference of serum creatinine (SCr) between two groups after cardiopulmonary bypass; (**C**) Real-time PCR show the difference of serum hsa-miR-494-3p between two groups after cardiopulmonary bypass in a time-dependent manner. N (the replicate for ELISA and Real-time PCR) = 3. Data represent the mean ± SEM. *P < 0.05, **P < 0.01 versus non-AKI.

According to KDIGO standard^26^ [serum creatinine increased by more than 0.3 mg/dL (26.5 mmol/L) within 48 h after cardiac surgery or more than 1.5 times higher than the preoperative baseline within 7 days after cardiac surgery]^26^, patients after cardiac surgery with cardiopulmonary bypass were divided into two groups: AKI and non-AKI patients (Fig. 1A). The changes of serum creatinine during perioperative period between the two groups were shown in Fig. 1B. The onset time of CSA-AKI was concentrated between the first day and the third day after operation. The serum creatinine of AKI patients in this study all recovered to the normal level around seven days after operation. Hsa-miR-494-3p in the serum of patients with CSA-AKI is elevated significantly at an hour after back into ICU, and returns to its baseline level at 12 h after back into ICU (Fig. 1C).

### Hsa-miR-494-3p increased inflammatory response in hypoxia-reoxygenation treated HK2 cells compared with the group treated with hypoxia

**Figure 2:**
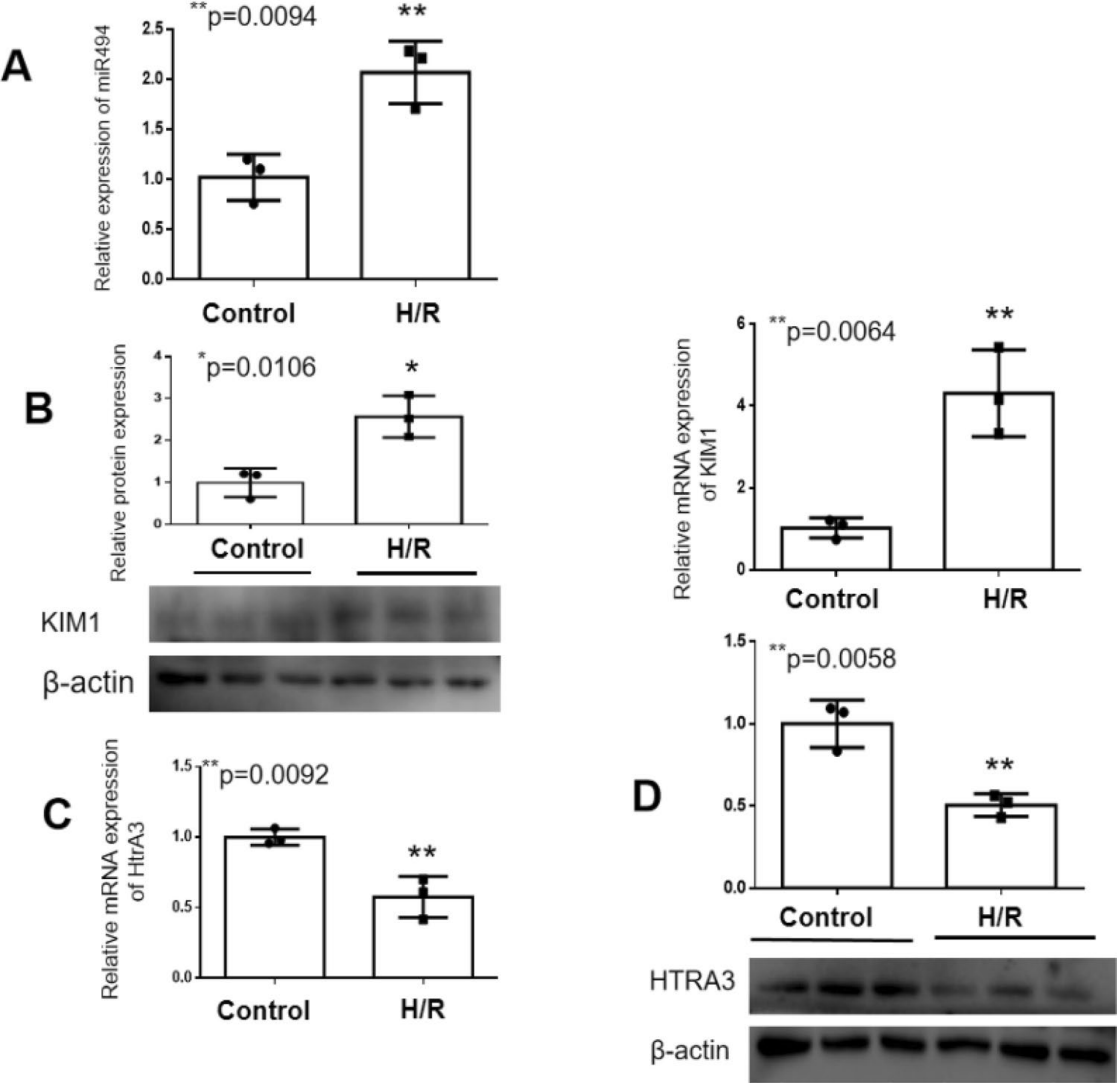
Effect of hsa-miR-494-3p on HK2 cells with or without hypoxia/reoxygenation. (**A**) Real-time PCR data show hypoxia/reoxygenation increased the expression of hsa-miR-494-3p; (**B**) Real-time PCR and Western blot data show hypoxia/reoxygenation increased the expression of KIM-1; (**C**) Real-time PCR data show hypoxia/reoxygenation decreased mRNA levels of HtrA3; (**D**) Western blot data show a lower expression of HtrA3 in the hypoxia-reoxygenation HK2 cells. N (the replicate for above experiments) = 3. *P < 0.05, **P < 0.01, versus control.

**Figure 3:**
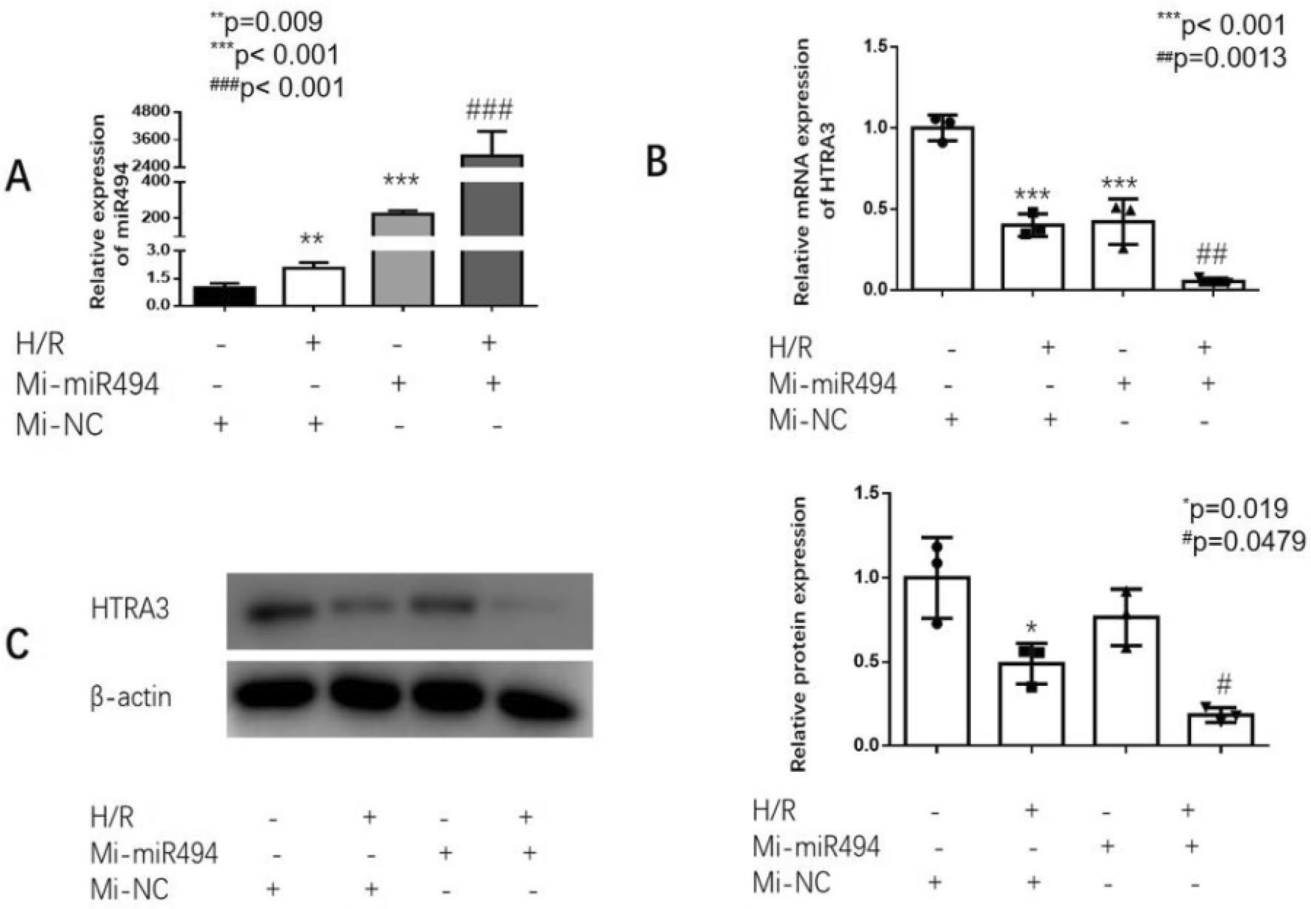
Hsa-miR-494-3p mimics further reduced the expression of HtrA3 by ischemia/reperfusion in HK2 cells. (**A**) Verification that hsa-miR-494-3p mimics were transfected in HK2 cells; (**B**) Real-time PCR data show hsa-miR-494-3p mimics further decreased mRNA levels of HtrA3; (**C**) Western blot data show hsa-miR-494-3p mimics has a lower expression of HtrA3 in HK2 cells, compared to the I/R. *P < 0.05, **P < 0.01, ***P < 0.001, versus control. N (the replicate for above experiments) = 3. ^#^P < 0.05, ^##^P < 0.01, ^###^P < 0.001, versus hypoxia/reoxygenation treatment group.

**Figure 4:**
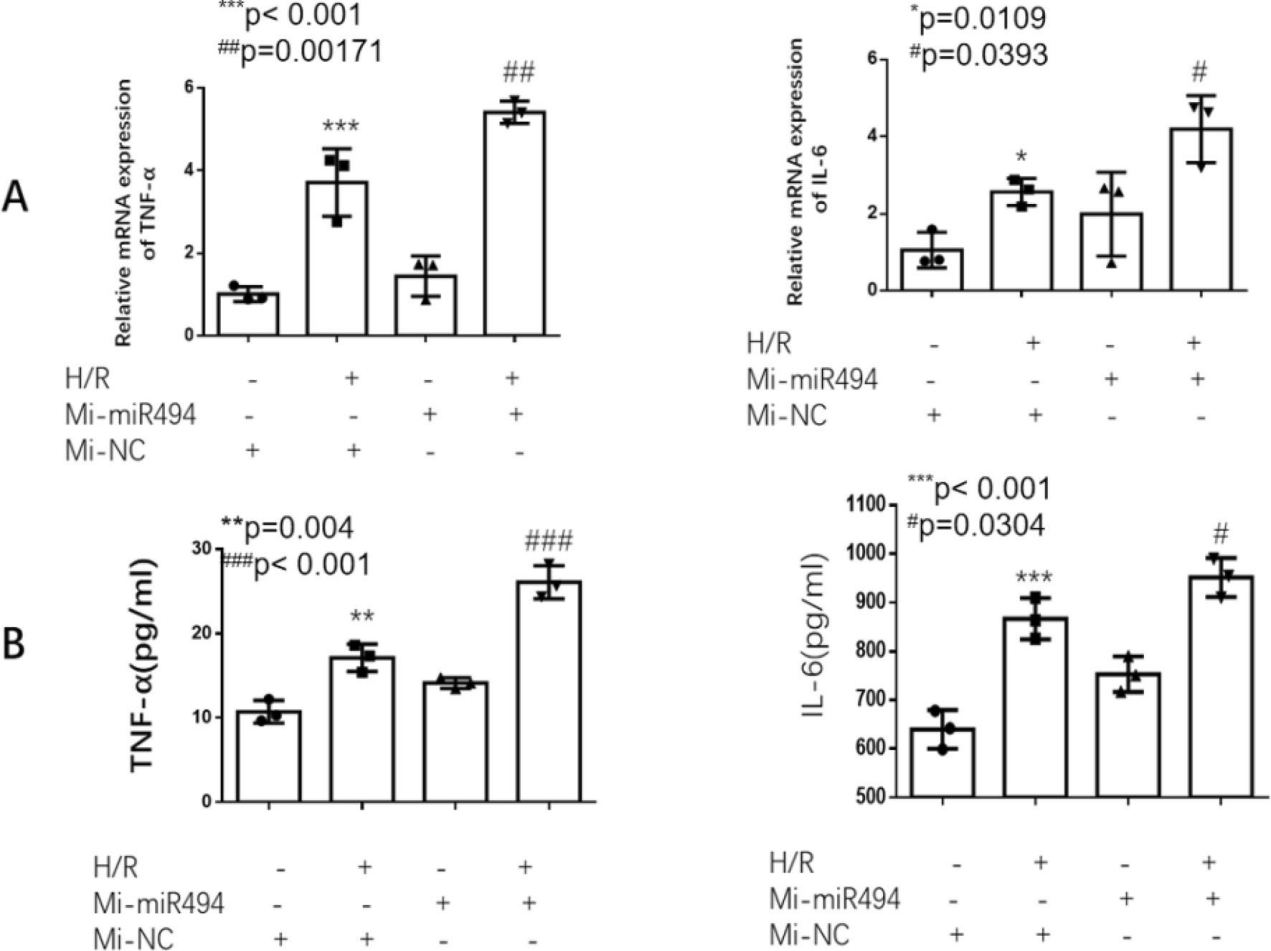
Hsa-miR-494-3p mimics increased ischemia/reperfusion-induced inflammatory response in HK2 cells. (**A**) Real-time PCR data show hsa-miR-494-3p mimics further increased ischemia/reperfusion-induced mRNA of TNF-α and IL-6; (**B**) ELISA data show overexpression of hsa-miR-494-3p significantly increased ischemia/reperfusion-induced TNF-α and IL-6. N (the replicate for above experiments) = 3. *P < 0.05, **P < 0.01, ***P < 0.001, versus control. ^#^P < 0.05, ^##^P < 0.01, ^###^P < 0.001, versus hypoxia/reoxygenation treatment group.

We use the public prediction platform (TargetScan Release 7.2, URL: http://www.targetscan.org/) and MicroRNA Target Prediction Database (miRDB, URL: http://mirdb.org/) to search 3′-UTR of HtrA3 which maybe a potential target of miR-494-3p. The expression of miR-494-3p went up significantly induced by hypoxia-reoxygenation, compared to the control group (Fig. 2A), and hypoxia/reoxygenation also induced KIM-1 up (Fig. 2B). However, the expression of HtrA3 in the hypoxia-reoxygenation HK2 cells was significantly lower than that in the control group (Fig. 2C,D). We overexpressed miR-494-3p by transfecting HK2 cells with miR-494-3p mimics (Fig. 3A). Overexpression miR-494-3p further decreased HtrA3 protein level inhibited by hypoxia/reoxygenation (Fig. 3B,C), and further increased ischemia/reperfusion-induced inflammatory response in HK2 cells. Inflammatory cytokines TNF-α and IL-6 increased after treatment of miR-494-3p mimics (Fig. 4A,B).

### Overexpression of HtrA3 downregulated the hypoxiareoxygenation-induced inflammatory response in HK2 cells, and the effect of overexpressed-miR-494-3p on cellular inflammatory responses is inhibited in HtrA3 overexpressing cells

**Figure 5:**
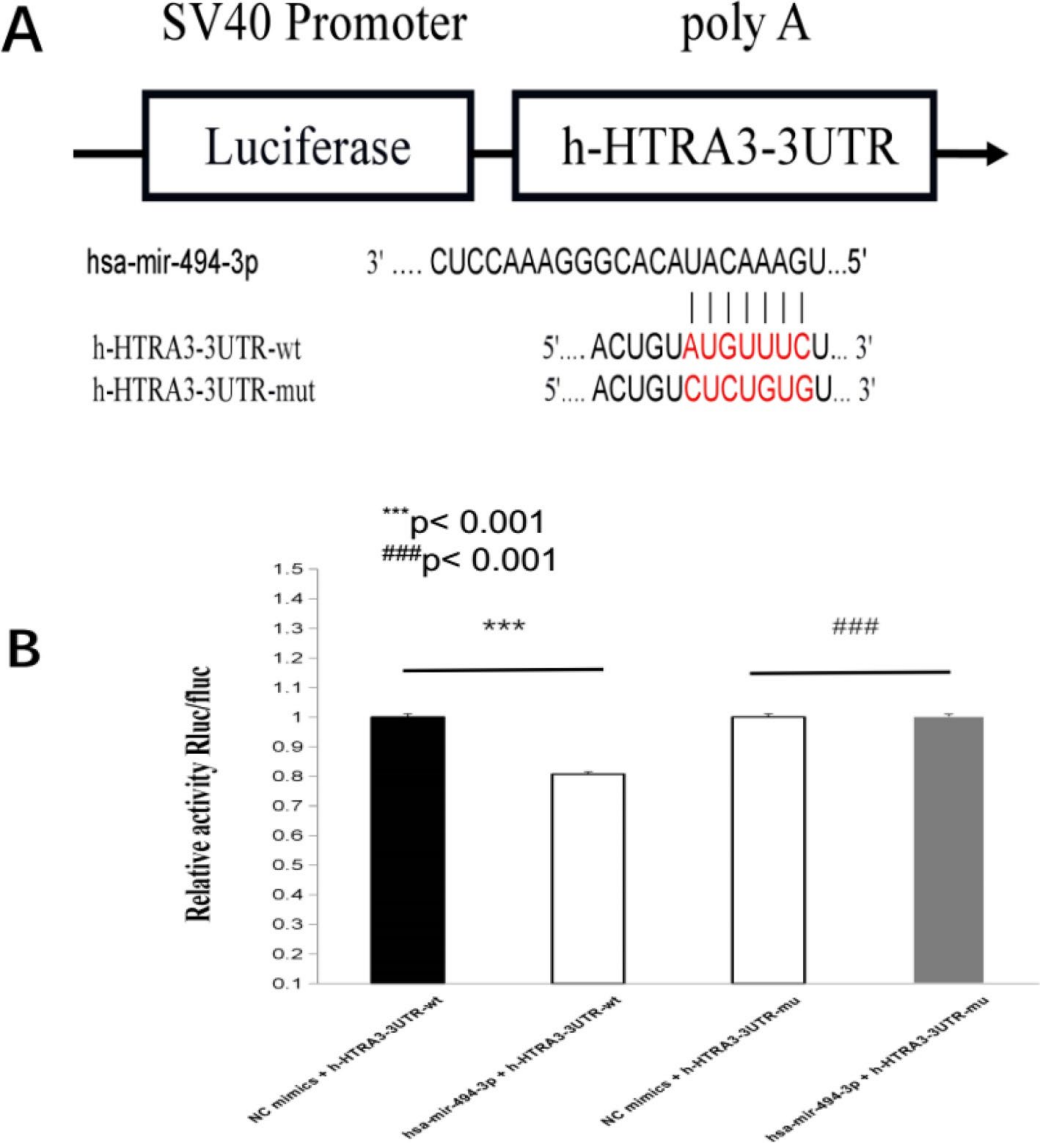
Hsa-miR-494-3p binds to 3′-UTR of HtrA3. (**A**) A target binding site between the 3′-UTR of HtrA3 and hsa-miR-494-3p; (**B**) Luciferase activity was significantly decreased in HK2 cells co-transfected hsa-miR-494-3p with wild-type 3′-UTR of HtrA3, but not in cells transfected with mutant 3′-UTR of HtrA3. N (the replicate for above experiment) = 3. *P < 0.05, **P < 0.01, ***P < 0.001, versus control. ^#^P < 0.05, ^##^P < 0.01, ^###^P < 0.001, versus hypoxia/reoxygenation treatment group.

**Figure 6:**
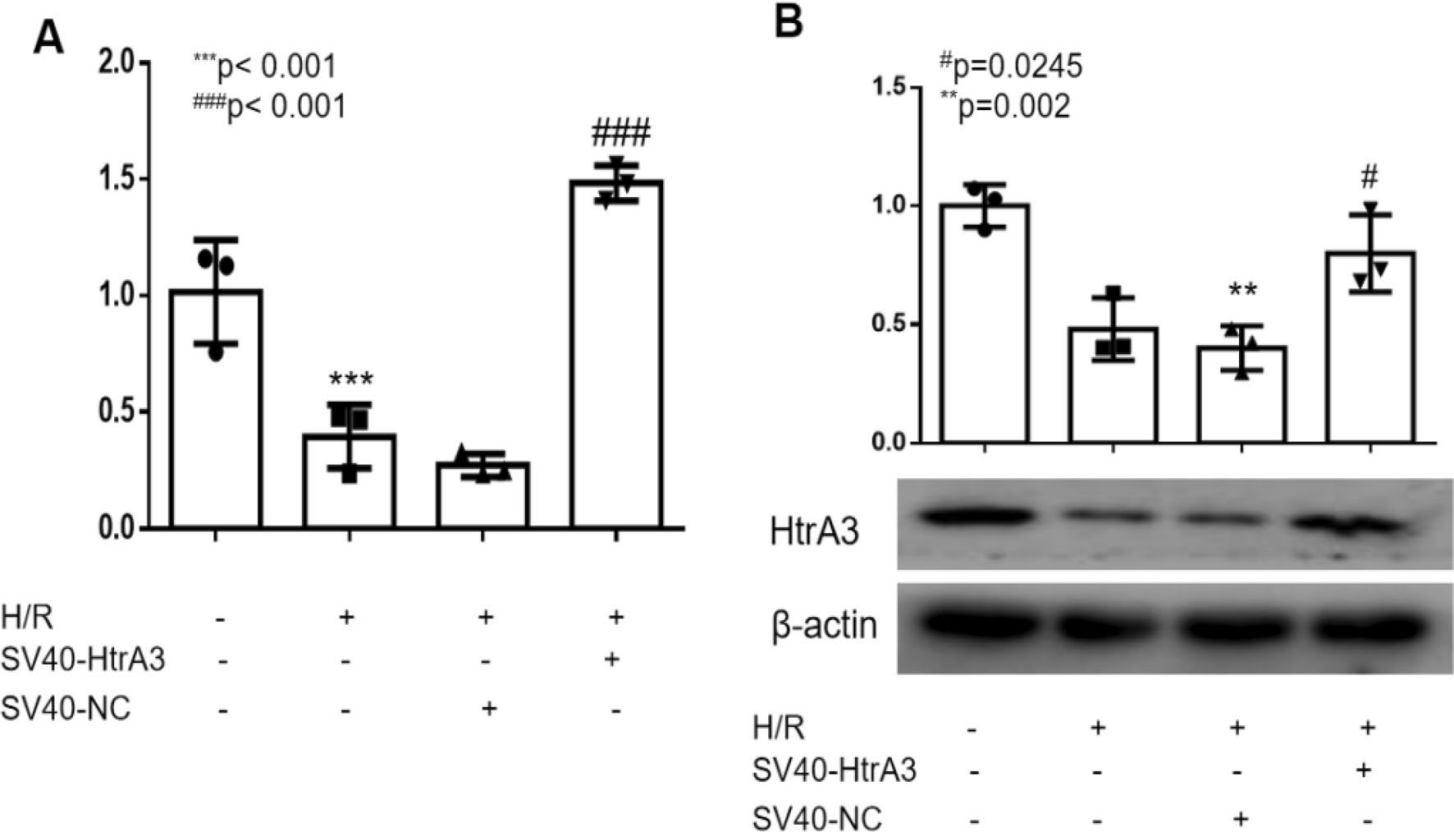
Overexpression of HtrA3 were transfected in HK2 cells. (**A**) Real-time PCR data show HtrA3 mRNA increased after HtrA3 were transfected in ischemia/reperfusion-induced HK2 cells; (**B**) Western blot data show HtrA3 protein level increased after HtrA3 were transfected in ischemia/reperfusion-induced HK2 cells. N (the replicate for above experiments) = 3. *P < 0.05, **P < 0.01, ***P < 0.001, versus control. ^#^P < 0.05, ^##^P < 0.01, ^###^P < 0.001, versus hypoxia/reoxygenation treatment group.

**Figure 7:**
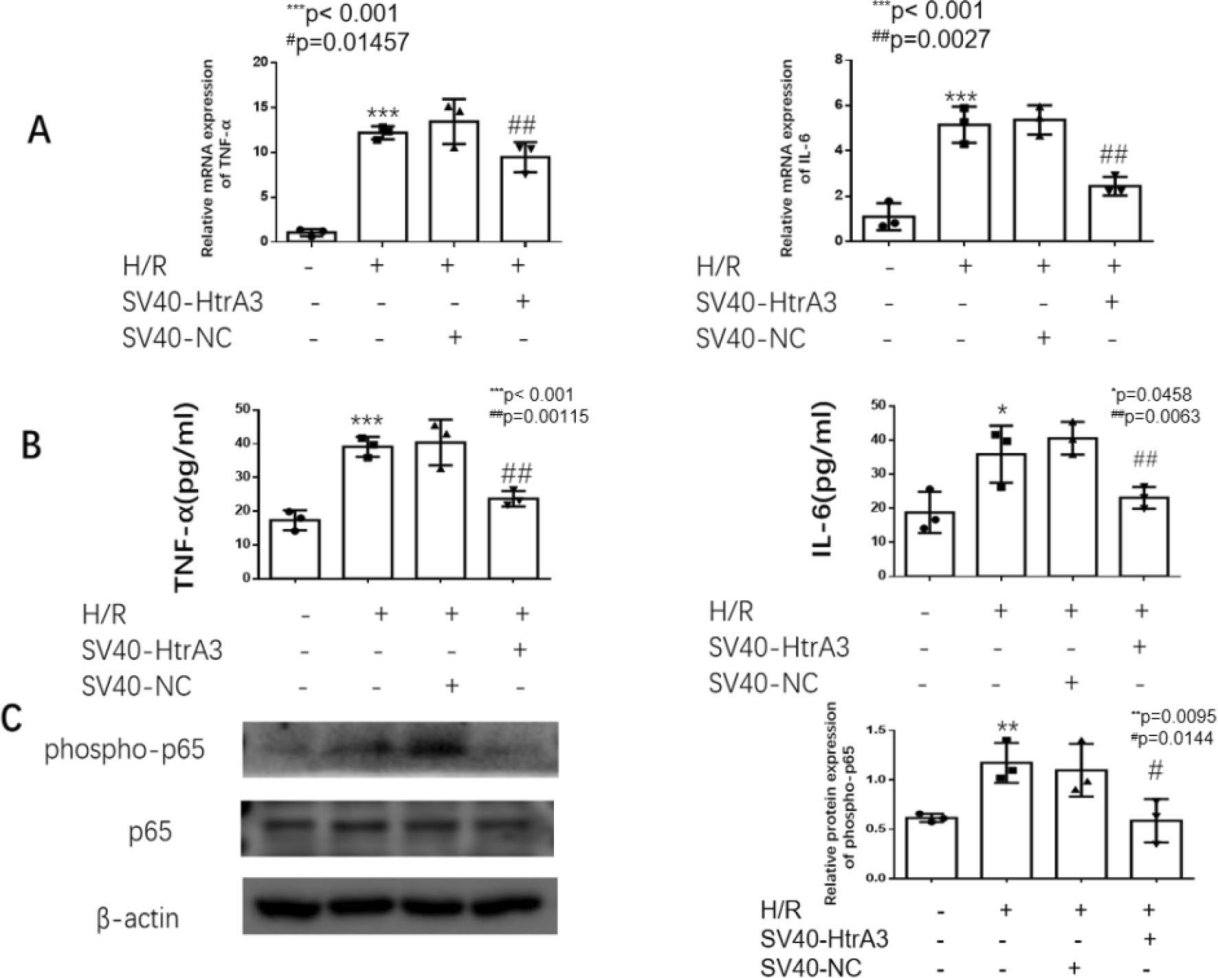
Overexpression of HtrA3 reduced ischemia/reperfusion-induced inflammatory response in HK2 cells. (**A**) Real-time PCR data show overexpression of HtrA3 decreased ischemia/reperfusion-induced mRNA of TNF-α and IL-6; (**B**) ELISA data show overexpression of HtrA3 decreased ischemia/reperfusion-induced TNF-α and IL-6 protein level; (**C**) Western blot data show overexpression of HtrA3 decreased ischemia/reperfusion-induced NF-κB phosphorylation. N (the replicate for above experiments) = 3. *P < 0.05, **P < 0.01, ***P < 0.001, versus control. ^#^P < 0.05, ^##^P < 0.01, ^###^P < 0.001, versus hypoxia/reoxygenation treatment group.

**Figure 8:**
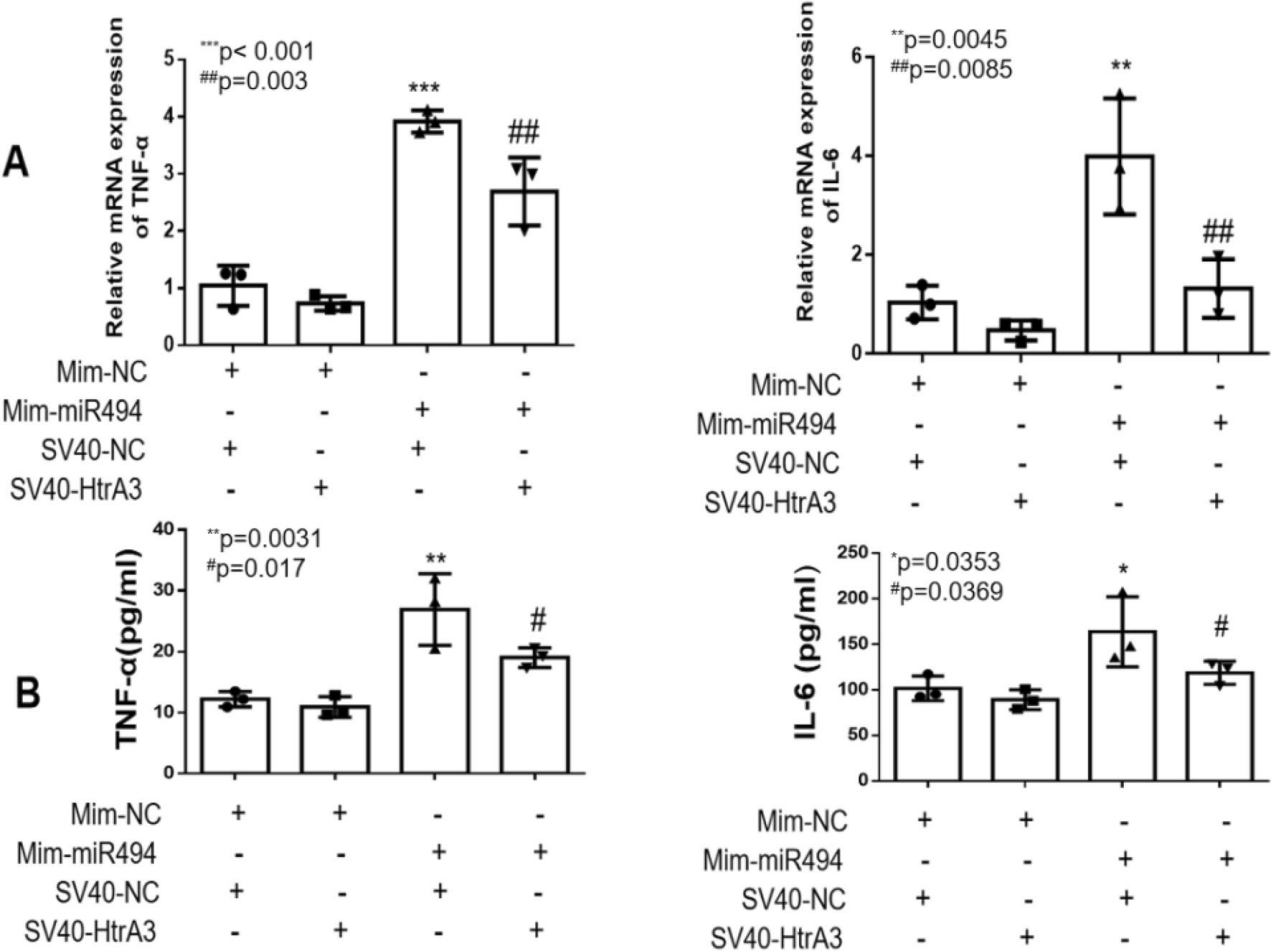
Hsa-miR-494-3p mimics-induced inflammatory response was inhibited by overexpression of HtrA3. (**A**) Real-time PCR data show overexpression of HtrA3 decreased hsa-miR-494-3p mimics-induced mRNA of TNF-α and IL-6; (**B**) ELISA data show overexpression of HtrA3 decreased hsa-miR-494-3p mimics-induced TNF-α and IL-6 protein level; N (the replicate for above experiments) = 3. *P < 0.05, **P < 0.01, ***P < 0.001, versus control. ^#^P < 0.05, ^##^P < 0.01, ^###^P < 0.001, versus SV40-NC group.

Luciferase report assay was performed to confirm the regulatory relationship between miR-494-3p and the 3′-UTR of HtrA3 (Fig. 5A,B). We overexpressed HtrA3 by transfecting HK2 cells with overexpression of HtrA3 (Fig. 6A,B). Overexpression of HtrA3 decreased ischemia/reperfusion-induced TNF-α and IL-6 (Fig. 7A,B) and NF-κB phosphorylation (Fig. 7C). HtrA3 downregulated the level of TNF-α and IL-6 significantly induced by miR-494-3p mimics treatment. However, miR-494-3p upregulated the level of TNF-α and IL-6 significantly and finally (Fig. 8A,B).

## Discussion

A kind of miRNAs can regulate the translation of various protein mRNAs. At present, the negative regulation mechanism of hsa-miR-494-3p on transcription factor 3 (ATF-3, a protein that protects kidney against stress response) protein synthesis has been clearly studied. miR-494-3p can be detected in renal tissue and urine, and it is upregulated during ischemia–reperfusion injury, which significantly inhibits the activation of ATF3, thus promoting the production of inflammatory mediators IL-6, MCP-1 and P-selectin in AKI model. Transfection of antisense miR-494-3p into mice improved renal injury induced by ischemia–reperfusion, reduced the production of pro-inflammatory cytokines and inhibited the apoptotic activity of caspase-3. This study also laid a solid foundation for the pathophysiological role of miR-494-3p in AKI model^25^. The study on cyclosporine (CsA)-induced nephrotoxicity showed kidney injury in mice was associated with an early increase in expression of miR-494, and it also explored tubular epithelial cell epithelial-mesenchymal transition induced by CsA toxicity resulted in the upregulation of miR-494 and a decrease in PTEN levels in vitro. miR-494 directly targeted Pten and negatively regulated its expression^27^.

In addition to targeting protein ATF3 and PTEN, our data show hsa-miR-494-3p attenuates gene HtrA3 transcription to increase inflammatory response in hypoxia/reoxygenation HK2. Like ATF3, HtrA3 proteins also play a protective role in ischemia–reperfusion kidney injury. The expression of HtrA3 protein may inhibit inflammatory response through nuclear factor-kappa B (NF-kappa B) signaling pathway which promotes renal inflammation, apoptosis and renal damage. The human HtrA family of ATP independent serine proteases (HtrA1, HtrA2, HtrA3, and HtrA4) are the key enzymes associated with pregnancy and closely related to the development and progression of many pathological events^28,29^, and they promote cell death in stress conditions and play a role as tumor suppressors. HtrA3 is a trimeric PDZ bearing propapoptotic serine protease, which is also involved in various diseases including pre-eclampsia and cancer^30^. A recent study on Endometrial cancer demonstrated hypoxia reduced the content of HtrA3, and silencing HtrA3 promoted Endometrial cancer cell migration, and as the degree of hypoxia increases in Endometrial cancer. Hypoxia leads to a decrease in HtrA3, which may promote cancer progression. These results indicated that HtrA3 plays an adaptive role in endometrial cancer in anoxic regions^31^. The human HtrA3 protease is involved in development and progress of many physiological events, including placentation, stimulation of apoptosis and considered as a tumor suppressor. The molecular mechanism of HtrA3’s function is unclear now, and knowledge of its cellular targets is limited^32^. Our study is the first to explore the role of HtrA3 in renal inflammation. It also explained that HtrA3 can reduce the renal inflammatory response induced by hsa-miR-494-3p, but cannot completely block or even reverse the kidney injury. So our results also suggested that hsa-miR-494-3p may play a complex network regulatory role in kidney injury (Supplementary information).

Studies have shown that urinary levels of miR-494-3p in AKI patients in comprehensive ICU increased before changes in serum creatinine and urea nitrogen^25^; however, there was no difference in serum samples, and their causes of AKI in the comprehensive ICU were inconsistent. Our study showed hsa-miR-494-3p is elevated in serum of CSA-AKI patients. Because some patients may have no urine in clinic, we did not test hsa-miR-494-3p in urine samples. However, the difference between these two studies in the detection of miR-494-3p in serum may be related to the time of kidney injury, and may also be related to prognosis. The pathogenesis of CSA-AKI is not clear^6^, and there are no effective preventive and therapeutic measures. At present, the indicators representing the loss of renal function cannot timely diagnose the occurrence of AKI. Because miRNAs regulated transcription of proteins, miRNAs should be more sensitive and earlier to be detected, compared with proteins, for example SCr, and miR-494-3p rose for 1 h after the surgery and returned to its baseline level at 12 h after the surgery. So hsa-miR-494-3p can be used as a molecular biological marker for early diagnosis of CSA-AKI patients. Hsa-miR-494-3p may participate in the early stage of the occurrence of CSA-AKI, and may also predict a good prognosis of CSA-AKI.

We showed that miR-494-3p was induced in response to hypoxia/reoxygenation injury which indicated AKI following ischemia–reperfusion injury. MiR-494-3p directly bound to 3′-UTR of HtrA3, which increased an anoxia-reperfusion induced inflammatory response. The expression of HtrA3 protein may inhibit inflammatory response through NF-kappa B signaling pathway. According to our knowledge, this is the first study that showed miR-494-3p also can increase renal inflammation by downregulation of HtrA3. The limitation of this study is just showing human miR-494-3p bound with the 3′-UTR of HtrA3, and lacking the protective role of HtrA3 on animal models of AKI. Collectively, Hsa-miR-494-3p may be used as an early diagnostic marker and a good prognostic predictor of CSA-AKI, and miR-494-3p/HtrA3 axis may serve as a novel therapeutic target for CSA-AKI.

## Materials and methods

### Detection of hsa-miR-494-3p in human serum

Adult patients undergoing on-pump valvular surgery or simultaneous coronary artery bypass grafting were selected. There were no chronic complications before operation and no organ damage except the kidney after operation. CSA-AKI diagnosis is based on the 2012 KDIGO criteria for improved global prognosis of kidney disease [serum creatinine increased by more than 0.3 mg/dL (26.5 mmol/L) within 48 h after cardiac surgery or more than 1.5 times higher than the preoperative baseline within 7 days after cardiac surgery]^26^.

### Target prediction analysis

We use the public prediction platform TargetScan (TargetScan Release 7.2, URL: http://www.targetscan.org/) and MicroRNA Target Prediction Database (miRDB, URL: http://mirdb.org/) to search potential 3′-UTR of the target mRNA that miR-494-3p binds.

### Cell culture, hypoxic treatment and transfection

Human kidney tubular epithelial cells (HK2) (provided by Prof. Hui Yao Lan in the Chinese University of Hong Kong) were cultured in DMEM/F12 with 5% FBS (Clark Bioscience, USA) medium in a humidified atmosphere of 5% CO2 at 37 °C. Medium was replaced every 2 days, and the cells were digested with 0.05% trypsin when the density of the cells reached 80–90%. HK2 cells were seeded in six-well plates or 96-well plates. When the density of cells reached 50–60% in 6-well plates, the experimental group cells were incubated overnight with serum-free medium. After that, HBSS solution was replaced and cultured in a hypoxic environment containing 1% O_2_, 94% N_2_ and 5% CO_2_ for 12 h. After hypoxia, the cells were reoxygenated in a complete medium with 21% oxygen for 3 h. The plasmid for HtrA3 was generated by amplifying complementary DNA (cDNA) coding for HtrA3 cDNA and inserting cDNA coding for HtrA3 into destination vectors by Gateway cloning (Genechem, Shanghai). The N terminal region of the HtrA3 coding region containing the predicted CARD domain was cloned into the SV40-HtrA3 vector by using restriction sites. HK2 cells were transfected with SV40-HtrA3 or microRNA-494 mimics by using Lipofectamine 2000 (Invitrogen, USA) for 24 h according to the manufacturer’s instructions. N (the replicate for these cell cultures, hypoxic treatments and transfections) = 3.

### RNA extraction and Real-time PCR

According to the manufacturer’s instructions, the total RNA was extracted from the HK2 cells by using TRIzol reagent (Invitrogen, USA). The abundance of miRNA was measured by quantitative real-time PCR (RT-qPCR) using One-Step miR RT-qPCR reagent kits and validated primers (Biomics, China) and the abundance of mRNA was measured by RT-qPCR using the system with SYBR-Green Master Mix (TaKaRa, Japan). The fold changes in the RNA expression were analyzed via the 2^−ΔΔCt^ method, and the level of the endogenous U6 RNA and β-actin performed as the normalized control. N (the replicate for these Real-time PCR analyses) = 3.

### Western blotting analysis

Total protein from cultured HK2 cells was harvested and lysed in RIPA lysis buffer (Beyotime, China) and protease inhibitor cocktail (Sigma, USA) for 30 min at 4 °C. The lysate was centrifuged for 15 min at 12,000 rpm and 4 °C, the upper supernatant was collected, and the protein was separated by 10% SDS-PAGE and transferred onto 0.45-μM polyvinylidene fluoride (PVDF) membranes (Millipore, Billerica, MA, USA)^33^. The membranes were blocked with 5% non-fat milk for 60 min and then washed in TBS-Tween 20 buffer for 15 min three times and incubated with specific primary antibodies at 4 °C overnight. Then, the membranes were washed three times with TBSTween 20 buffer and incubated with secondary antibody, horseradish peroxidase-conjugated anti-mouse antibody or anti-rabbit antibody (ZSGB-BIO, 1:10,000), at 25 °C for 1 h, and then developed onto the chemiluminescence Western blotting detection system using an ECL chemiluminescent kit (ECL-plus, Thermo Scientific). Quantitative densitometric analyses of immunoblotting images were performed using Image J software (NIH, Bethesda, USA). And the experiment was repeated three times^33^. Primary antibodies were as follows: HtrA3 (Abcam, USA, 1:800), P65 (CST, USA), p-p65 (CST, USA), and β-actin (Bioss, Beijing, China). N (the replicate for these Western blotting analyses) = 3.

### ELISA assay

The culture medium of cells and serum were collected, centrifuged and supernatant was obtained. The OD value of each sample (wavelength: 450 nm) was determined by micrometer according to the instructions, and the contents of IL-6, TNF-α were calculated according to the obtained standard curve (Neobioscience, China). N (the replicate for these ELISA assays) = 3.

### Luciferase reporter assay

The HtrA3 3′UTR carrying a putative miR-494-3p binding site was generated by PCR and then cloned into pMiR-report vector to establish the luciferase reporter constructs HtrA3-wild-type and HtrA3-mutated-type. Subsequently, cells were co-transfected with the reporter constructs HtrA3-wild-type or HtrA3-mutated-type and miR-494-3p vector or scramble using transfection reagent (Hanbio, shanghai, China) according to the manufacturer’s instructions. For determination of the luciferase activity, reporter assays were done using the dual-luciferase assay system^34^. N (the replicate for these Luciferase reporter assays) = 3.

### Statistical analysis

Data are expressed as the mean ± SEM. Statistical significance was analyzed by two-tailed unpaired *t* test or one-way analysis of variance (ANOVA), followed by Tukey post hoc tests using GraphPad Prism 6 software^35^.

### Ethics approval and consent to participate

This study was approved by the Ethics Committee of the First Affiliated Hospital of Anhui Medical University and informed consent of patients was obtained, and all experimental protocols used in the present study were in compliance with the guidelines of the Declaration of Helsinki.

## Supplementary Information

Supplementary Information.

## Data Availability

The data that support the findings of this study are available on request from the corresponding author. The data are not publicly available due to privacy or ethical restrictions.
