# Supplementary material for: Hsa-miR-494-3p attenuates gene HtrA3 transcription to increase inflammatory response in hypoxia/reoxygenation HK2 Cells
Source: Sci Rep. 2021 Jan 18;11:1665. doi: 10.1038/s41598-021-81113-x (PMC7814133; doi:10.1038/s41598-021-81113-x)

**Hsa-miR-494-3p** **attenuates gene HtrA3 transcription to increase inflammatory response in hypoxia/ reoxygenation HK2 Cells**

Qian Gong^1,*^, Zhi-ming Shen^1,*^, Zhe Sheng^1^, Shi Jiang^1^, Sheng-lin Ge^1^*

1. Department of Cardiovascular Surgery, The First Affiliated Hospital of Anhui Medical University, Hefei, 230022, China

*These authors contributed equally to this work.

*Corresponding author:

Sheng-Lin Ge, Professor of cardiology,

Department of Cardiovascular Surgery, the First Affiliated Hospital of Anhui Medical University, Hefei, Anhui, China. 230022

Email: [shenglin_ge@163.com](mailto:shenglin_ge@163.com)


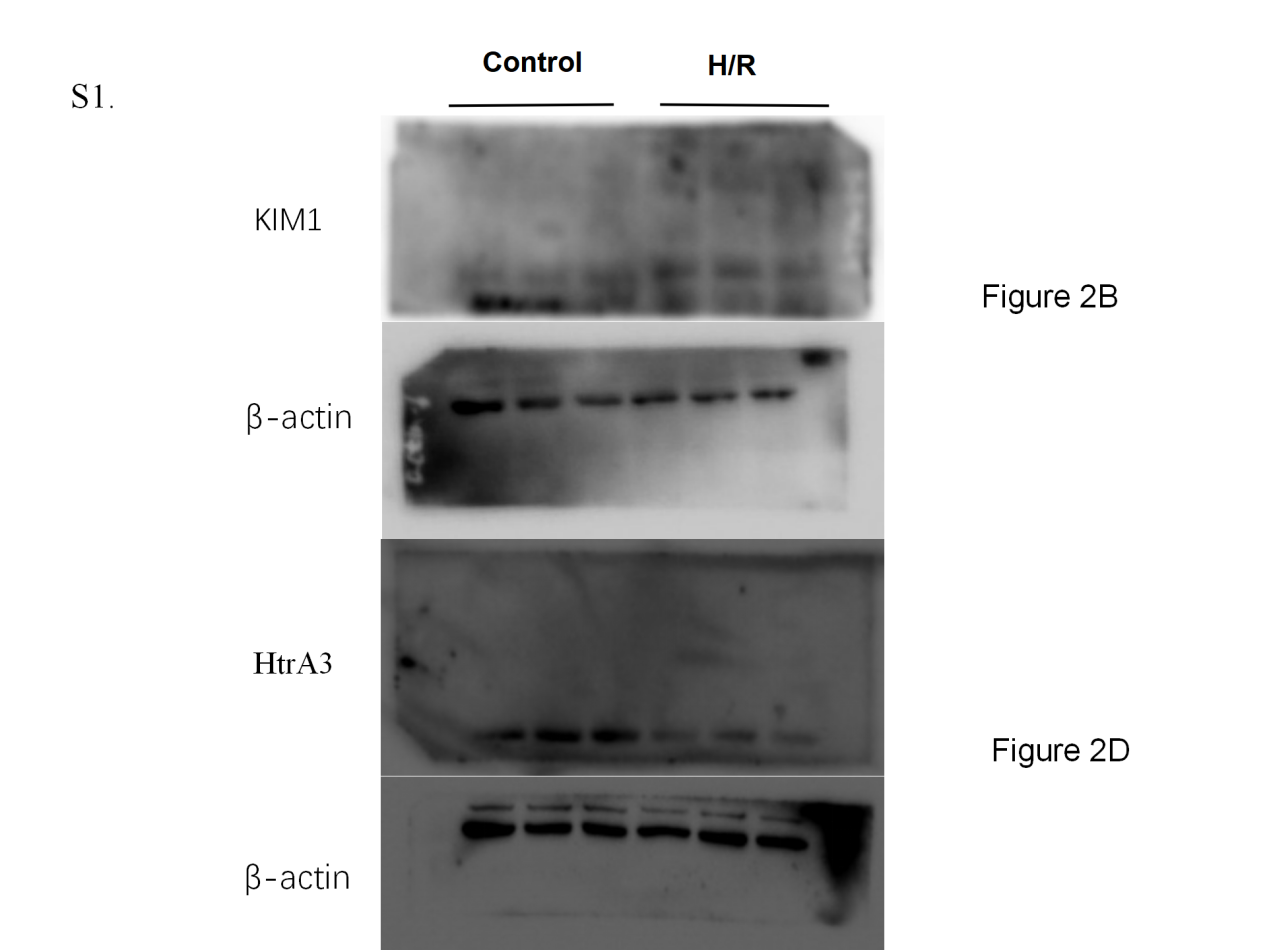


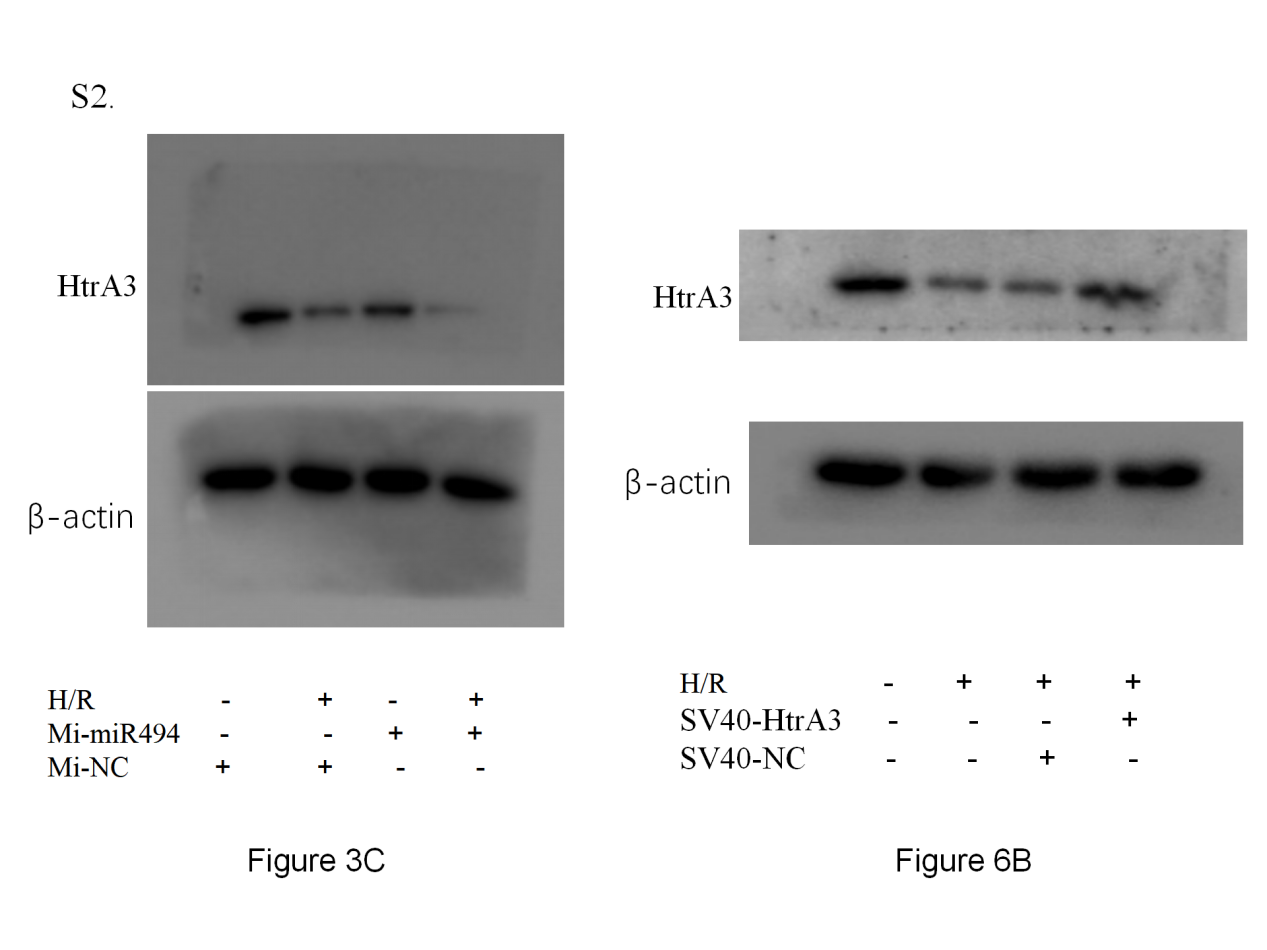


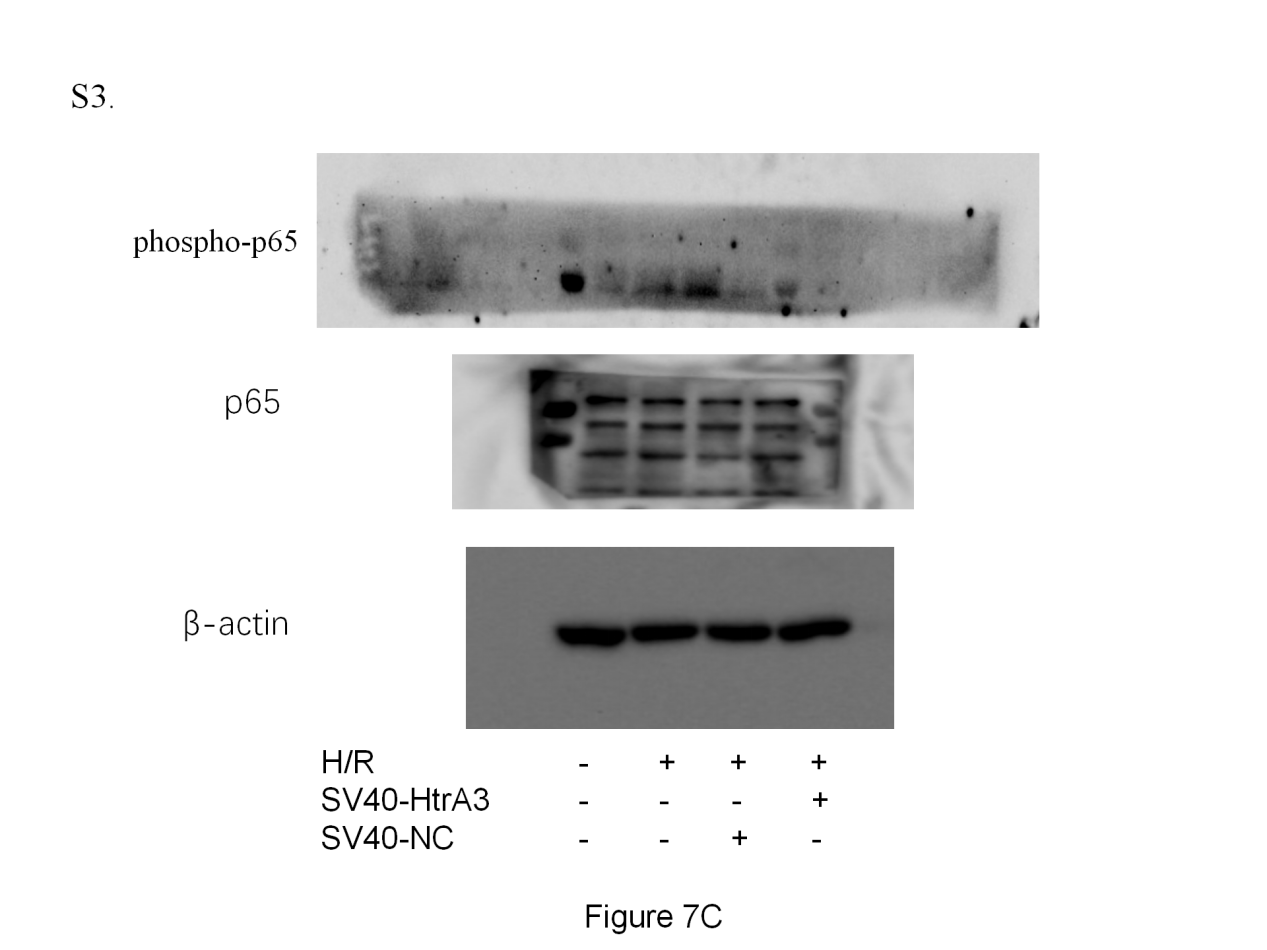

Supplement: Supplementary file 1 — Supplementary Information. [file 41598_2021_81113_MOESM1_ESM.docx]
